# Research on the energy absorption performance of diamond structures with axial and transverse porosity gradients under different load-bearing angles

**DOI:** 10.3389/fbioe.2026.1947630

**Published:** 2026-10-28

**Authors:** Zhenjia Wang, Anas Hassan, Xuzhe Qiu, Yunfei Lan, Yongtao Lyu, Yadong Liu

**Affiliations:** 1 Department of Orthopedics, Dalian Municipal Central Hospital Affiliated to Dalian University of Technology, Dalian, China; 2 DUT-BSU Joint Institute, Dalian University of Technology, Dalian, China; 3 School of Mechanics and Aerospace Engineering, Dalian University of Technology, Dalian, China

**Keywords:** additive manufacturing, diamond lattice structure, energy absorption performance, gradient porosity, mechanical metamaterials

## Abstract

**Background:**

In biomedical, aerospace, automotive and marine engineering, there is an increasing demand for high energy absorption behaviour in structures. Triply periodic minimal surface (TPMS) structures, such as Diamond lattices, have emerged as promising solutions due to their light weight nature and excellent energy absorption performance, and thus they have attracted significant research interest.

**Methods:**

This study explores the energy absorption characteristics and deformation behavior of Diamond structures with varying porosity distributions under different loading angles. Uniform and gradient Diamond structures with axial and transverse porosity distributions were generated. Quasi-static compression tests were performed on the uniform structures, while FE simulations were used to investigate the mechanical and energy-absorption responses of the uniform and gradient configurations. Quasi-static compression tests and finite element (FE) simulations were used to analyze the changes in energy absorption characteristics and deformation/damage modes during the compression process.

**Results:**

The results show that loading orientation and porosity-gradient direction strongly influence the energy-absorption response of Diamond structures. For the uniform structure, the SEA increased to approximately 8 kJ/kg at a loading angle of 30°, representing an increase of about 33% compared with the 0° and 15° configurations, while the corresponding plateau stress reached approximately 105 MPa. When the loading angle increased to 45°, the SEA decreased to approximately 4 kJ/kg because of earlier densification. Axial-gradient structures exhibited increased plateau stress and slightly enhanced SEA, but their maximum energy-absorption efficiency remained below approximately 35%, compared with about 40% for the uniform structures. Transverse-gradient structures showed plateau stresses comparable to those of the uniform structure with the corresponding average porosity, but tended to densify earlier, resulting in reduced SEA.

**Conclusion:**

These findings provide valuable insights into the design and optimization of Diamond structures for improved energy-absorption performance under different loading conditions and offer mechanical design guidance that may be relevant to the future development of TPMS structures for biomedical applications.

## 1 Introduction

Lattice structures are becoming increasingly important in the fields of biomedical engineering, aerospace, automotive, and marine engineering. Lattice structures exhibit excellent mechanical (Zhang et al., 2024a; Chen et al., 2012), thermal (Lu et al., 1998; Saghir et al., 2025; Zeng et al., 2024), electrical (Huddy et al., 2023), and acoustic properties (Ibrahim et al., 2018; Swanson, 1984) and have demonstrated significant potential in lightweight design (Yan et al., 2014; Maheshwaraa et al., 2007; Ataollahi, 2023) and energy absorption (Ormiston and Srinivas Sundarram, 2024; Shi et al., 2023; Zhang et al., 2022; Günaydın et al., 2022; Zhang et al., 2021). Among these, triply periodic minimal surface (TPMS) lattice structures have garnered considerable attention due to their high strength, high energy absorption efficiency, and unique zero mean curvature (Zhang et al., 2024b; Wan et al., 2024; Zhong et al., 2021). Their continuous and smooth surfaces facilitate uniform stress distribution, thereby enhancing load-bearing capacity and enabling smooth fracturing (Yoo, 2015; Yoo, 2014). To date, TPMS structures have been applied in various fields, including atmospheric water harvesting devices (Gado et al., 2024; Gado and Ookawara, 2023), orthopedic implants (Barber et al., 2021; Yuan et al., 2019; Bobbert et al., 2017), efficient thermal management (Yetik et al., 2024), and noise control (Saatchi et al., 2023). Among the numerous TPMS configurations, the Primitive, Gyroid, and Diamond structures have become research hotspots due to their high manufacturability.

The performance of TPMS structures is significantly influenced by their porosity (Pałka et al., 2016; Rajagopalan and Robb, 2006). However, the porous nature of these structures may also compromise their mechanical properties (Dunand, 2004). When it is necessary to simultaneously meet biological, mechanical, or thermal requirements, porosity becomes particularly critical. One example is bone implants, which must satisfy both biological and mechanical properties (Iamsamang and Naiyanetr, 2021; Yoo, 2011). As heterogeneous and anisotropic nanocomposite materials, bone implants exhibit a multi-level structural organization of their main components from the macroscopic to the nanoscale. Therefore, to enhance mechanical properties and biological functions, the development of TPMS structures with gradient porosity has become a research focus. Porous materials with gradient porosity possess several advantageous characteristics. Their customizable internal pore surfaces can be utilized for organic, inorganic, or biological coatings (Song et al., 2021). Moreover, internal pores can be filled with biocompatible materials, degradable materials, or even living cells (Yan et al., 2025). These features endow TPMS structures with significant potential for advancing high-performance porous materials. However, compared to uniform porous materials, research on porosity gradient materials based on TPMS structures faces additional challenges.

The finite element (FE) method is widely employed to predict the mechanical properties of TPMS lattices, including compressive strength, energy absorption, stress distribution, and anisotropy. These analyses facilitate a deeper understanding of the deformation behavior and underlying mechanisms of such structures. To date, most FE studies have focused on uniform TPMS structures. For instance, Shin et al. investigated the mechanical properties of TPMS structures with varying porosities and reported that the Primitive structure exhibits good stability and fluid permeability (Shin et al., 2012). Song et al. designed Primitive and Gyroid structures with four different porosities and used FE analysis to evaluate the stress distribution in tooth root analog implants (Song et al., 2021). Lai et al. employed FE analysis and computational fluid dynamics to assess the mechanical properties of a new type of functionally graded bone scaffold with different porosities, demonstrating superior mechanical properties and mass transfer capabilities compared to traditional scaffolds (Lai et al., 2025). Despite these advances, FE analysis of TPMS structures with gradient porosity remains underexplored.

Earlier studies have emphasized that the energy absorption performance of TPMS structures has become a global research focus. However, systematic studies on the energy absorption behavior of TPMS structures with linear porosity gradients along the axial and transverse directions under different load-bearing angles are still lacking. Such studies are essential to examine the stability and flexibility of these structures under multi-directional loads. A detailed analysis of the energy absorption performance of TPMS structures with porosity gradients under many different load-bearing angles, and elucidation of the underlying performance trends, would provide important guidance for practical applications and lay the theoretical basis for the performance-oriented optimization of TPMS structures with respect to energy absorption in the future.

Different TPMS topologies exhibit distinct mechanical responses and anisotropic behaviors due to differences in surface curvature, internal connectivity and load-transfer pathways. The Gyroid lattice generally shows low sensitivity to loading orientation and maintains relatively stable energy-absorption capacity under multi-directional compression. By contrast, the Diamond topology possesses pronounced orientation-dependent mechanical properties; its deformation mode, plateau stress and specific energy absorption change significantly with loading angle, making it well-suited for investigating the coupling effect between loading orientation and porosity-gradient design. The Primitive structure exhibits size-dependent transverse performance and differs substantially from Diamond in deformation mechanisms (Skibar et al., 2026). It should be emphasized that the Diamond structure is not universally superior to Gyroid or Primitive in terms of compressive strength, plateau stress or specific energy absorption. Each topology has its own merits. The present work focuses on exploring the coupled effects of porosity gradient and loading angle within a fixed Diamond topology, rather than performing a comprehensive performance comparison among different TPMS families.

This study investigates the compression behavior, deformation characteristics, and energy absorption performance of the Diamond structure with linear gradient porosities along the axial and transverse directions under different load-bearing angles using FE analysis. In addition, the relationship between deformation mode and energy absorption capacity was elucidated by analyzing the changes in energy absorption associated with different deformation modes. The results provide mechanical design insights into the orientation-dependent behavior of gradient TPMS structures and may offer a reference for their future development in mechanically demanding applications, including potential biomedical scenarios.

## 2 Materials and methods

### 2.1 Materials

Ti-6Al-4V was selected as the base material for the Diamond structure. This material has a density of 4,500 kg/m^3^, a Young’s modulus of 110.0 GPa, a Poisson’s ratio of 0.3 and a yield stress of 480 MPa. The plasticity curve parameters quoted are shown in Table 1. These data were obtained from tensile tests reported in the literature (Yin et al., 2020).

**TABLE 1:** Plasticity curve parameters of Ti-6Al-4V.

Plastic strain	Plastic stress (MPa)
0	480
0.0057	495
0.0498	516
0.1004	536
0.1509	558
0.2014	582
0.2518	608
0.2956	644

### 2.2 Design of diamond structure

Considering that the anisotropy of a structure significantly affects its mechanical properties during the elastic stage, in this study the Diamond structure is selected for investigation, because it exhibits distinct anisotropic characteristics. The Diamond structure was computationally designed using the open-source Flatt Pack Generator (Version 3.35, University of Nottingham, United Kingdom) (Maskery et al., 2022). As shown in Figure 1, the dimensions of the single cell were 3 mm × 3 mm × 3 mm. a cubic specimen with dimensions of 12 mm × 12 mm × 12 mm was constructed based on the single cell. A unit-cell size of 3 mm and a 4 × 4 × 4 arrangement were adopted consistently for all configurations in the present study. This fixed geometric setting was selected to maintain consistency with the manufactured specimen dimensions and to ensure that the effects of loading orientation and porosity-gradient direction could be evaluated without introducing unit-cell size or specimen size as additional variables. It should be noted that the present study does not systematically investigate unit-cell-size or specimen-size effects. Therefore, possible finite-size and boundary effects should be considered when extrapolating the present results to substantially larger TPMS structures. The conclusions of the present study should therefore be interpreted within the geometric scale investigated here. The homogeneous Diamond structure with 70% porosity was rotated along the X-axis by four angles (0°, 15°, 30°, and 45°), resulting in four kinds of different models. In addition, porosity gradients were introduced along the axial (z-axis) and transverse (y-axis) directions, respectively. In both cases, the porosity varied linearly from 60% to 80%. The axial-gradient structure therefore represents a porosity variation parallel to the nominal compression direction, whereas the transverse-gradient structure represents a porosity variation perpendicular to the nominal compression direction, which in turn generated another 8 different computational models. Thus, a total of 12 different models were generated in this paper. The typical porosity of human trabecular bone is approximately 70%, ranging from 50% to 90% across different anatomical sites. The porosity range of 60%–80% was selected symmetrically around the 70% reference porosity used for the uniform Diamond structure. This design allows a pronounced spatial porosity gradient to be introduced while maintaining an overall average porosity close to 70%, thereby enabling the influence of porosity redistribution to be evaluated without substantially changing the total material volume. The 60%–80% range was adopted as a representative gradient amplitude for the present comparative study rather than as an optimized or universally applicable design range. Other gradient amplitudes were not systematically investigated and therefore remain an important topic for future parametric optimization.

**FIGURE 1:**
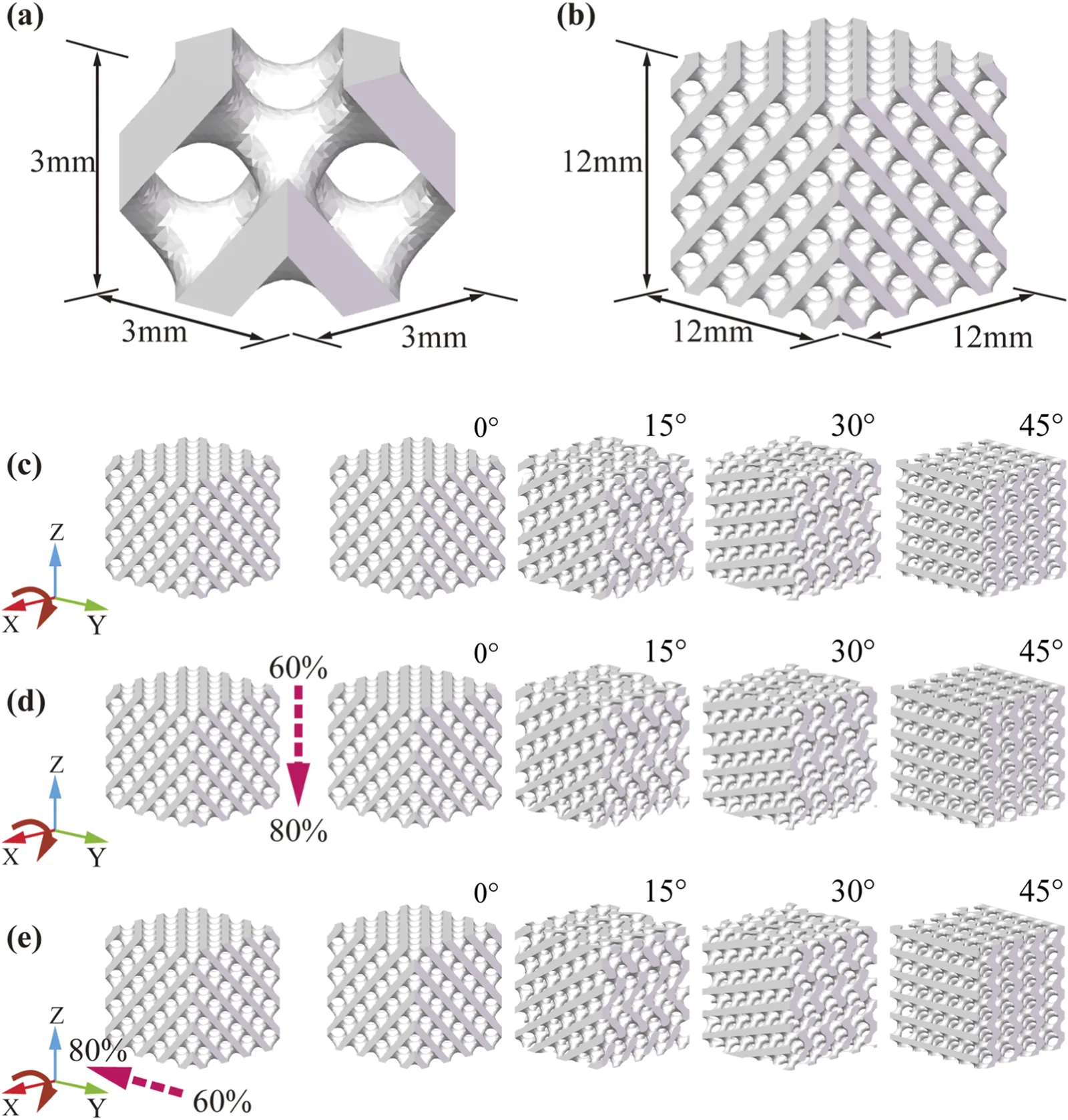
The model of the diamond structures: **(a)** cell size; **(b)** the size of 4 × 4 × 4 model; Schematic diagrams of **(c)** uniform diamond structures, **(d)** axial gradient diamond structures and **(e)** transverse gradient diamond structures for different load-bearing angles.

To ensure a fair comparison between the uniform and gradient structures, the actual solid volumes of the generated geometric models were calculated. The solid volume of the uniform Diamond structure with 70% porosity was 518.4448 mm^3^, while those of the axial-gradient and transverse-gradient structures were 517.6393 mm^3^ and 517.7688 mm^3^, respectively. Considering an overall specimen envelope volume of 1728 mm^3^, the corresponding overall porosities were 69.9974%, 70.0440%, and 70.0365%, respectively, as summarized in Table 2. The deviations in solid volume between the axial-gradient and transverse-gradient structures and the uniform structure were only 0.1554% and 0.1304%, respectively. These small differences confirm that the three configurations possess essentially equivalent material volumes and overall porosities. Therefore, the differences in energy-absorption performance can be primarily attributed to the spatial redistribution of porosity rather than to differences in total material volume.

**TABLE 2:** Geometric equivalence of the uniform and gradient Diamond structures.

Structure	Porosity design	Solid volume (mm3)	Overall porosity (%)	Volume difference relative to uniform (%)
Uniform diamond	70%	518.4448	69.9974	—
Axial-gradient diamond	60%–80%	517.6393	70.0440	0.1554
Transverse-gradient diamond	60%–80%	517.7688	70.0365	0.1304

The uniform Diamond structure with 70% porosity was selected as the reference configuration because this value corresponds to the midpoint of the 60%–80% porosity range adopted for both the axial and transverse gradient designs. This choice provides an approximately equivalent baseline in terms of overall porosity and material volume, allowing the effect of spatial porosity redistribution to be evaluated more directly. It should be noted that the 70% porosity adopted here represents a reference design parameter within the scope of the present study rather than a universally optimal porosity for biomedical implants.

### 2.3 Quasi-static compression test

The ability of the Diamond structure to absorb energy, is strongly governed by its stress–strain response. The Ti-6Al-4V Diamond TPMS specimens were fabricated by selective laser melting (SLM) using an SLM Solutions SLM280 system at the Key Laboratory of Thermal Energy and Power Technology. Gas-atomized spherical Ti-6Al-4V powder with a particle-size range of 15–45 μm was used as the feedstock. An ytterbium-doped fiber laser with a power of 180 W and a scanning speed of 1,200 mm/s was employed. The layer thickness and hatch spacing were set to 30 μm and 120 μm, respectively. High-purity argon was supplied to the build chamber, and the oxygen concentration was maintained below 100 ppm to minimize oxidation. The build platform was preheated to 200 °C. A chessboard scanning strategy was adopted, with the scanning direction rotated by 67° between adjacent layers to mitigate residual stress accumulation. The specimens were tested in the as-built condition, without post-fabrication heat treatment or surface finishing prior to quasi-static compression testing.

The stress–strain response of the Diamond structure was determined under quasi-static compressive tests using a SANS compression testing machine rated with a load capacity of up to 50.0 kN. The vertical compression rate was set to 0.5 mm/min, and the experimental setup is shown in Figure 2a. The upper and lower platens were adjusted to remain parallel to each other, and the specimen was positioned centrally on the plateau prior to testing. Sensors were attached to the compression plates to collect data on reaction forces and displacement during the compression process. The collected data were then analyzed by computer to produce force-displacement curves. Each uniform Diamond configuration was tested using three independent specimens ( n=3 ). The experimental stress–strain response was obtained by averaging the results of the three specimens, and the experimental variability was quantified using the standard deviation. The resulting mean stress–strain curves were used to calculate the energy-absorption efficiency curves shown in Figure 3. Experimental variability in the quantitative mechanical and energy-absorption parameters presented in Figure 4 is represented by error bars corresponding to ± one standard deviation based on the three independent tests.

**FIGURE 2:**
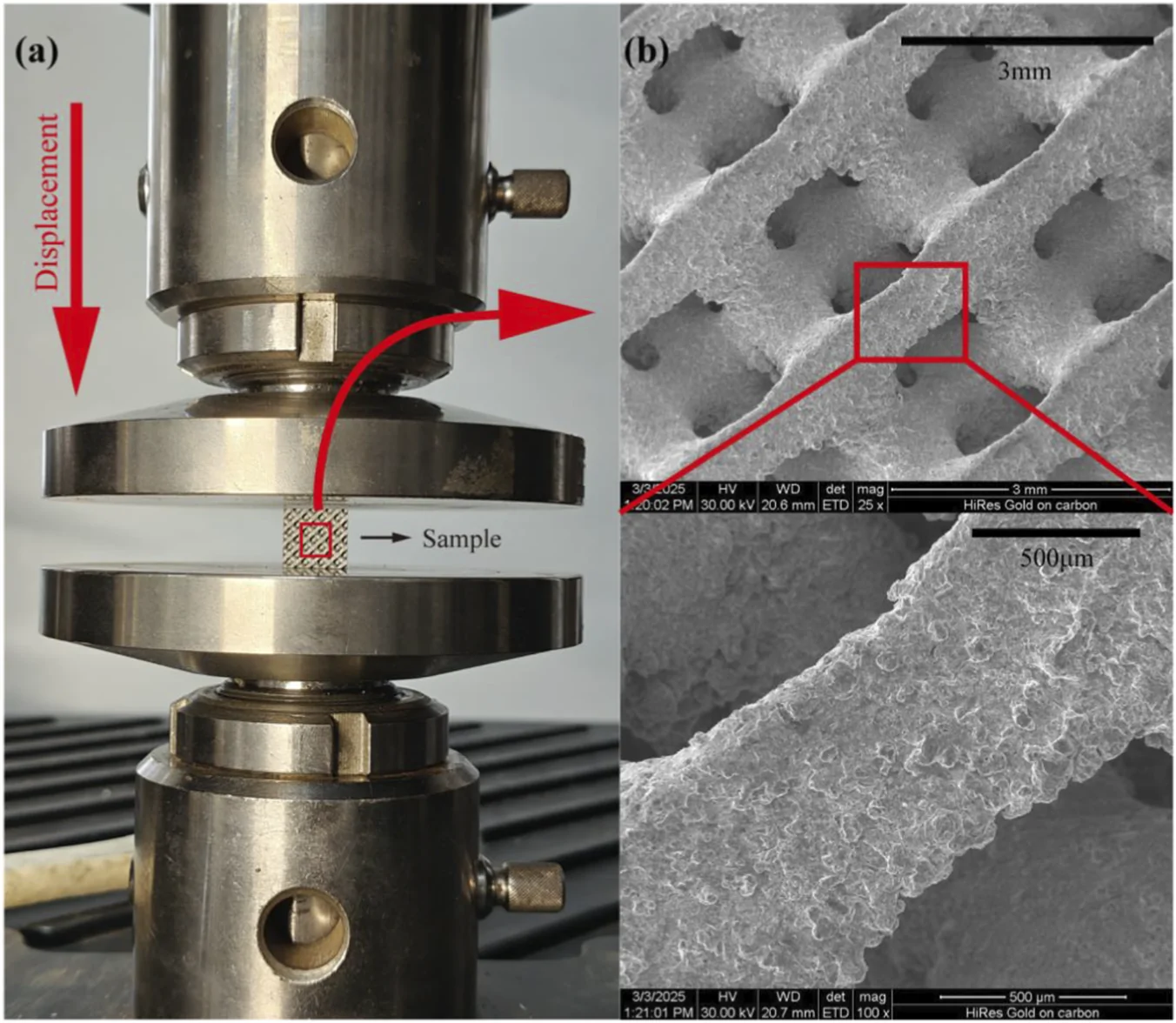
**(a)** Setup of the quasi-static compression test. **(b)** Surface topography of the metal printed sample.

**FIGURE 3:**
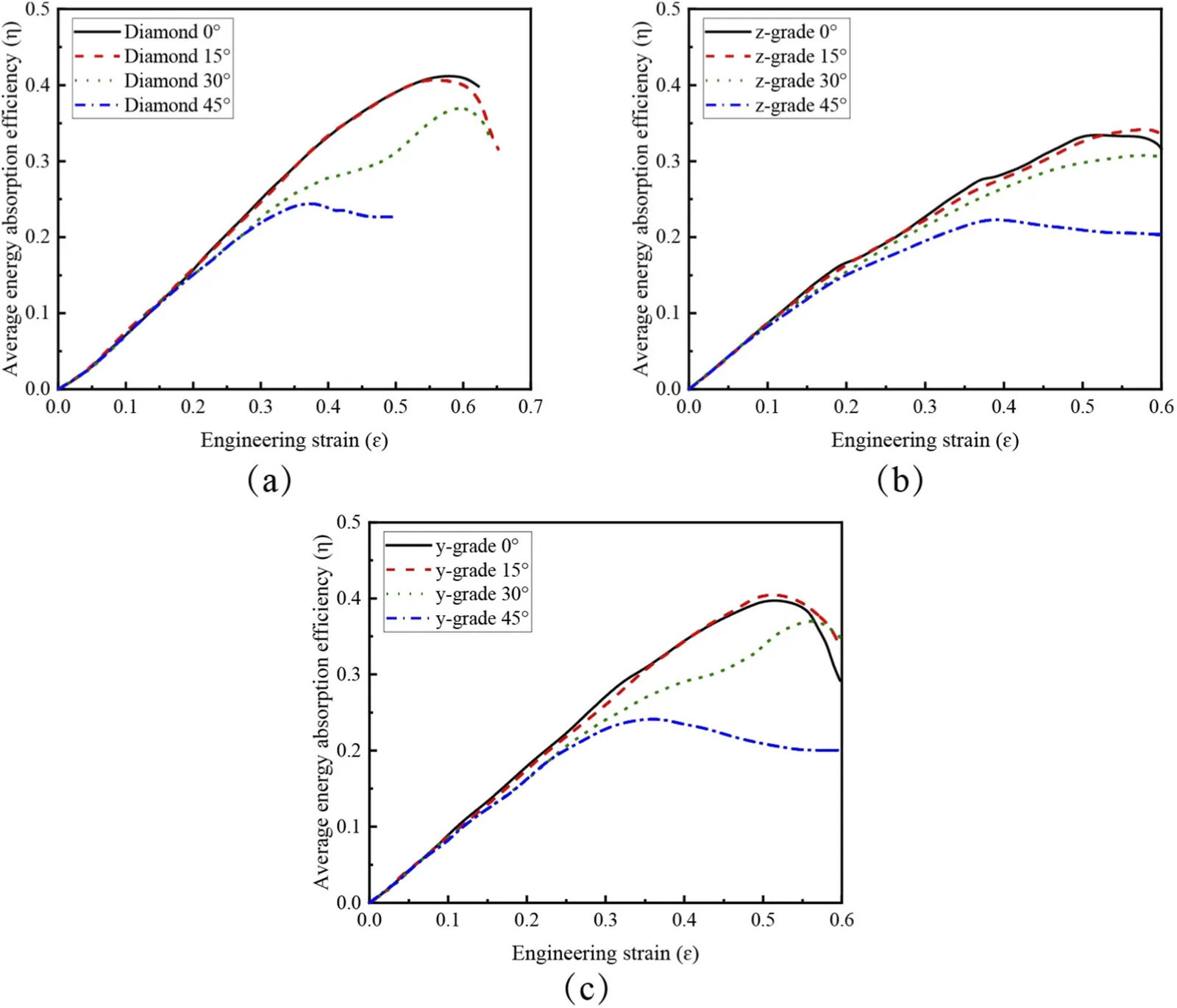
Energy absorption efficiency curves for **(a)** uniform diamond, **(b)** axial gradient diamond and **(c)** transverse gradient diamond structures. For the uniform diamond structures, the experimental energy-absorption efficiency curves were calculated from the corresponding mean stress–strain curves obtained from three independent tests (n = 3).

**FIGURE 4:**
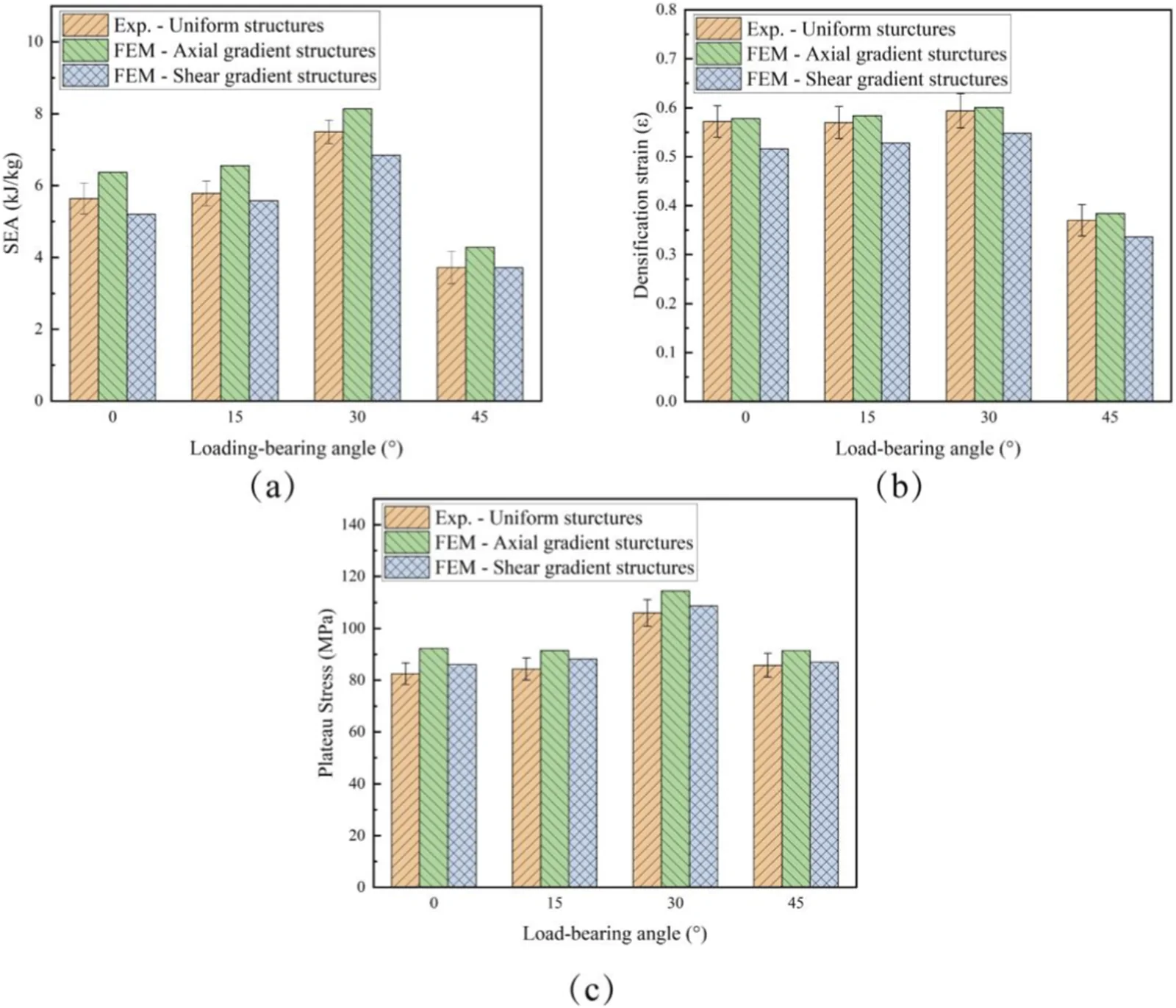
**(a)** Special energy absorption, **(b)** densification strain and **(c)** plateau stress of Diamond structures. Error bars represent ± one standard deviation from three independent tests ( n=3 ).

To verify the accuracy and reliability of the experimental results, a quality assessment was conducted on the samples after fabrication using scanning electron microscopy (SEM). Figure 2b shows the sample surface, which appears slightly rough. This minor roughness is likely due to the adhesion of Ti-6Al-4V powder layers during printing or the texture formed naturally during the cooling process (Yu et al., 2025). SEM observations were used to qualitatively evaluate the surface morphology of the fabricated specimens. However, μCT-based three-dimensional reconstruction was not performed in the present study; therefore, internal geometric deviations, including local wall-thickness variation and pore-shape deviations, could not be quantitatively assessed. This limitation should be considered when comparing the experimental results with FE predictions based on the nominal CAD geometry.

### 2.4 Simulation of FE quasi-static compression

#### 2.4.1 Constitutive and damage models

Quasi-static compression simulations were performed using ABAQUS/Explicit 2021. Ti-6Al-4V was modeled as an isotropic elastic–plastic material. The Young’s modulus and Poisson’s ratio were E=110 GPa and ν=0.30 , respectively, and the initial yield stress was 480 MPa. The post-yield response was defined using the tabulated true stress–equivalent plastic strain data listed in Table 1, with an isotropic hardening assumption. The von Mises yield criterion was adopted as Equation 1:

$$f=q-\sigma_{y}\left({\bar{\varepsilon }}^{pl}\right)=0$$ (1)

where $q=\sqrt{3s:s/2}$ is the von Mises equivalent stress, s is the deviatoric stress tensor, ${\bar{\varepsilon }}^{pl}$ is the equivalent plastic strain, and $\sigma_{y}\left({\bar{\varepsilon }}^{pl}\right)$ is the current yield stress obtained by piecewise-linear interpolation of the tabulated plasticity data. An associated plastic-flow rule was employed.

Strain-rate-dependent plastic hardening was not considered because the present study focuses on quasi-static compression. Although an accelerated loading rate was adopted in the explicit analysis for computational efficiency, the kinetic-to-internal-energy ratio was monitored throughout the simulation and remained below 5% during the main compression stage, indicating that inertial effects were sufficiently limited.

Material damage initiation was described using the Johnson–Cook damage criterion, as shown in Equation 2:

$${\bar{\varepsilon }}_{D}^{pl}=\left[D_{1}+D_{2}\exp\left(D_{3}\eta \right)\right]\left[1+D_{4}\ln\left(\frac{{\dot{\bar{\varepsilon }}}^{pl}}{{\dot{\varepsilon }}_{0}}\right)\right]\left(1+D_{5}T^{*}\right)$$ (2)

where η=−p/q is the stress triaxiality, p is the hydrostatic pressure, ${\dot{\bar{\varepsilon }}}^{pl}$ is the equivalent plastic-strain rate, ${\dot{\varepsilon }}_{0}$ is the reference strain rate, and $T^{*}$ is the normalized temperature. The Johnson–Cook damage parameters for Ti-6Al-4V were $D_{1}=-0.68$ , $D_{2}=0.73$ , $D_{3}=-0.25$ , and $D_{4}=D_{5}=0$ (Kadkhodapour et al., 2015). Therefore, the strain-rate and temperature terms in the damage-initiation equation do not contribute under the adopted parameter set.

Damage accumulation before initiation was evaluated as Equation 3:

$$\omega_{D}=\sum \frac{\Delta {\bar{\varepsilon }}^{pl}}{{\bar{\varepsilon }}_{D}^{pl}}$$ (3)

and damage initiation occurred when $\omega_{D}=1$ . After damage initiation, an energy-based damage-evolution law was employed to describe progressive stiffness degradation. The mode-I fracture energy was calculated as Equation 4:

$$G_{f}=\frac{\left(1-\nu^{2}\right)K_{Ic}^{2}}{E}$$ (4)

where $K_{Ic}=49\text{ MPa}\sqrt{m}$ (Cain et al., 2015), E=110 GPa, and ν=0.30 , giving $G_{f}=19.86\;N/\text{mm}$ . The damaged stress was expressed as a Equation 5:

$$\sigma =\left(1-D\right)\bar{\sigma }$$ (5)

where $\bar{\sigma }$ is the effective stress tensor of the undamaged material and D is the scalar damage variable. Element deletion was activated when the damage variable reached complete degradation ( D=SDEG=1 ). The output variables DUCTCRT, SDEG, STATUS, PEEQ, and stress were extracted to distinguish damage initiation, progressive material degradation, element deletion, and structural collapse.

#### 2.4.2 Mesh convergence analysis

The Diamond structures were discretized using eight-node linear hexahedral reduced-integration elements (C3D8R). Mesh convergence was evaluated using the uniform Diamond structure with 70% porosity under 0° compression. Characteristic element sizes of 0.5, 0.3, and 0.2 mm were examined, corresponding to approximately $1.00\times 10^{6}$ , $2.80\times 10^{6}$ , and $5.00\times 10^{6}$ elements, respectively. The resulting plateau stresses were 75, 79, and 81 MPa, while the corresponding SEA values were 5.2, 5.6, and 5.7 kJ/kg, respectively.

Refining the mesh from 0.3 to 0.2 mm changed the plateau stress and SEA by only 2.47% and 1.75% as shown in Table 3, respectively, whereas the number of elements increased by approximately 78.6%. A variation below 3% between the two finest meshes was regarded as acceptable convergence. Therefore, a characteristic element size of 0.3 mm was selected as a compromise between numerical accuracy and computational cost. Because element deletion may introduce additional mesh sensitivity, the same element size and damage formulation were consistently applied to all structural configurations.

**TABLE 3:** Mesh sensitivity analysis of the 70% porosity uniform Diamond structure under 0° loading.

Mesh size (mm)	Element numbe	Plateau stress (MPa)	SEA (kJ/kg)
0.5	1.00 × 106	75	5.2
0.3	2.80 × 106	79	5.6
0.2	5.00 × 106	81	5.7

#### 2.4.3 Contact, loading and quasi-static control

The specimen was positioned between two analytical rigid plates. The lower plate was fully constrained, whereas all degrees of freedom of the upper plate were constrained except for translation along the loading direction. General contact in ABAQUS/Explicit was used to account for both specimen–plate contact and self-contact of the Diamond structure during progressive collapse. Hard contact was adopted in the normal direction, while a penalty friction formulation with a friction coefficient of 0.08 was applied in the tangential direction.

A prescribed displacement of 7.2 mm was applied to the upper rigid plate, corresponding to 60% nominal compressive strain for the 12-mm-high specimen, as shown in Figure 5. The total analysis time was 1.0 s, corresponding to an average numerical loading velocity of 7.2 mm/s. A smooth-step amplitude was adopted to reduce artificial inertial effects caused by abrupt acceleration and deceleration. The stable explicit time increment was automatically determined by ABAQUS based on the smallest element dimension and material wave speed.

**FIGURE 5:**
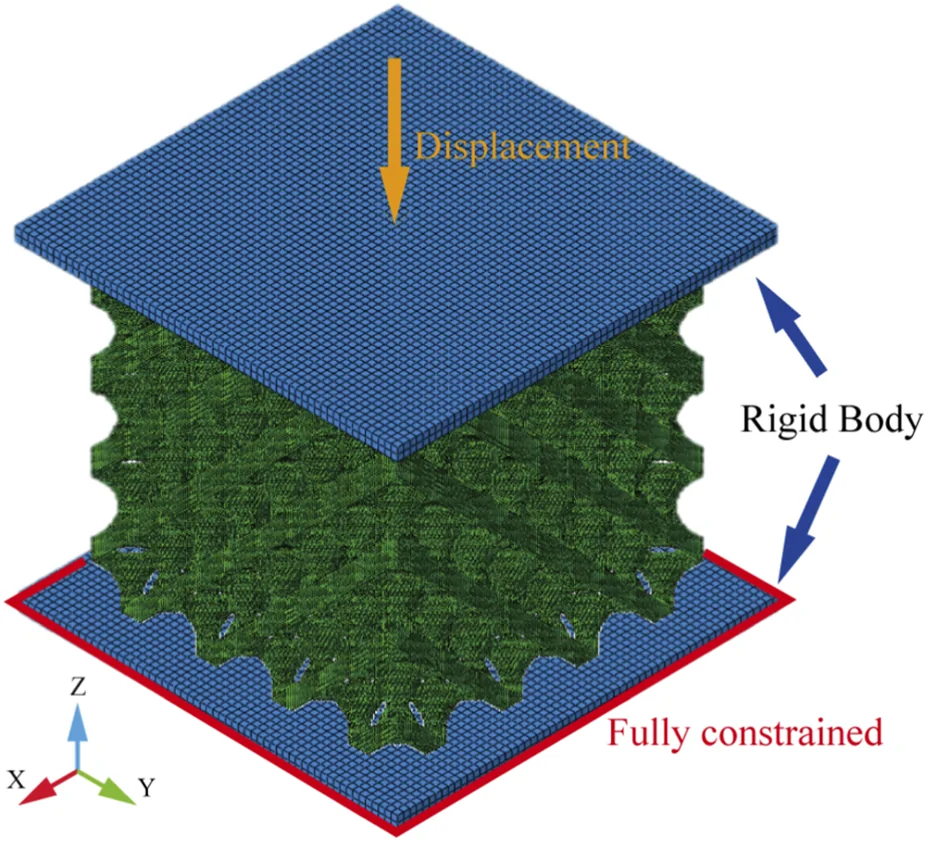
FE model and the boundary condition for the quasi-static compression simulation.

The numerical loading rate was intentionally accelerated relative to the experimental rate to reduce computational cost. Because the constitutive plasticity model was rate-independent and the kinetic-to-internal-energy ratio remained below 5% during the main compression stage, the accelerated loading was considered to retain a quasi-static response.

No mass scaling was used. The histories of kinetic energy, internal energy, artificial strain energy, and total energy were monitored throughout the analysis to assess the quality of the explicit quasi-static solution.

### 2.5 Energy absorption capability

The force–displacement data obtained from testing is transformed into corresponding stress–strain curves. The efficiency of energy absorption, expressed as a function of structural strain, is given in the following Equation 6.

$$\eta \left(\varepsilon \right)=\frac{w}{\sigma \left(\varepsilon \right)}$$ (6)

where $\eta \left(\varepsilon \right)$ indicates the energy absorption efficiency of the structure; $w=\int_{0}^{\varepsilon }\sigma \left(\varepsilon \right)d\varepsilon$ indicates the absorbed energy density accumulated during compression up to strain ε ; $\sigma \left(\varepsilon \right)$ represents the stress related to the strain.

The stress–strain behavior of the Diamond structure under quasi-static loading can be divided into three clearly distinguishable phases: an initial elastic phase, followed by a stress plateau phase and a final densification phase (Qiu et al., 2024). Also known as the plastic stage, the stress plateau phase is characterized by stress remaining nearly constant or rising gradually with increasing strain. During this phase, the internal stress of the structure is referred to as the plateau stress ( $\sigma_{pl}$ ), as described in Equation 7.

$$\sigma_{pl}=\frac{\int_{\varepsilon_{s}}^{\varepsilon_{e}}\sigma \left(\varepsilon \right)d\varepsilon }{\varepsilon_{e}-\varepsilon_{s}}$$ (7)

In this study, the onset strain of the plateau region, $\varepsilon_{s}$ , was determined using the 0.2% offset method. Specifically, a line parallel to the initial linear-elastic portion of the stress–strain curve was shifted by a strain offset of 0.002. The intersection between this offset line and the experimental or numerical stress–strain curve was taken as the onset of yielding and defined as $\varepsilon_{s}$ . The same procedure was applied consistently to all experimental and FE curves to ensure comparability of the calculated plateau stresses. The densification strain, $\varepsilon_{d}$ , was determined from the onset of the rapid stress increase associated with structural densification, using the same criterion for all configurations.

Specific energy absorption (SEA) describes the capacity of a structure to absorb energy under external loading or impact conditions. The SEA is expressed by the given Equation 8.

$$SEA=\frac{w_{v}}{M}$$ (8)

Energy-absorption efficiency and specific energy absorption characterize different aspects of structural performance. Energy-absorption efficiency is a dimensionless parameter describing the effectiveness of energy absorption relative to the instantaneous stress level at a given strain, whereas SEA represents the total energy absorbed per unit mass of the structure and is expressed in kJ/kg. Therefore, a structure with a high SEA does not necessarily exhibit the highest energy-absorption efficiency.

Where M denotes the mass per unit volume of the structure, measured in kg/m^3^; $w_{v}$ denotes the total absorbed energy per unit volume of the structure, measured in kJ/m^3^.

## 3 Results and discussion

### 3.1 Validation of the FE prediction

To ensure the credibility of the material data used, particularly the accuracy of the plasticity curve data, the simulation parameters were validated in this section. To evaluate the reliability of the FE model under different loading orientations, uniform Diamond structures with 70% porosity at loading angles of 0° and 45° were selected for experimental validation. The 70% porosity was selected as the reference condition because it corresponds to the midpoint of the 60%–80% porosity range adopted for the gradient structures, thereby providing a consistent baseline for evaluating the effect of spatial porosity redistribution. It should be noted that 70% represents a reference design parameter within the scope of the present study rather than a universally optimal porosity for biomedical implants. The corresponding numerical stress–strain responses were compared with the experimental results. The 0° configuration represents the baseline compression condition, whereas the 45° configuration was selected to assess the applicability of the numerical model under a strongly rotated loading orientation. As shown in Figure 6, the numerical predictions reproduce the overall elastic response, plateau behavior, and densification trend observed experimentally for both loading conditions, supporting the applicability of the FE framework to the orientation-dependent analysis of the uniform Diamond structures.

**FIGURE 6:**
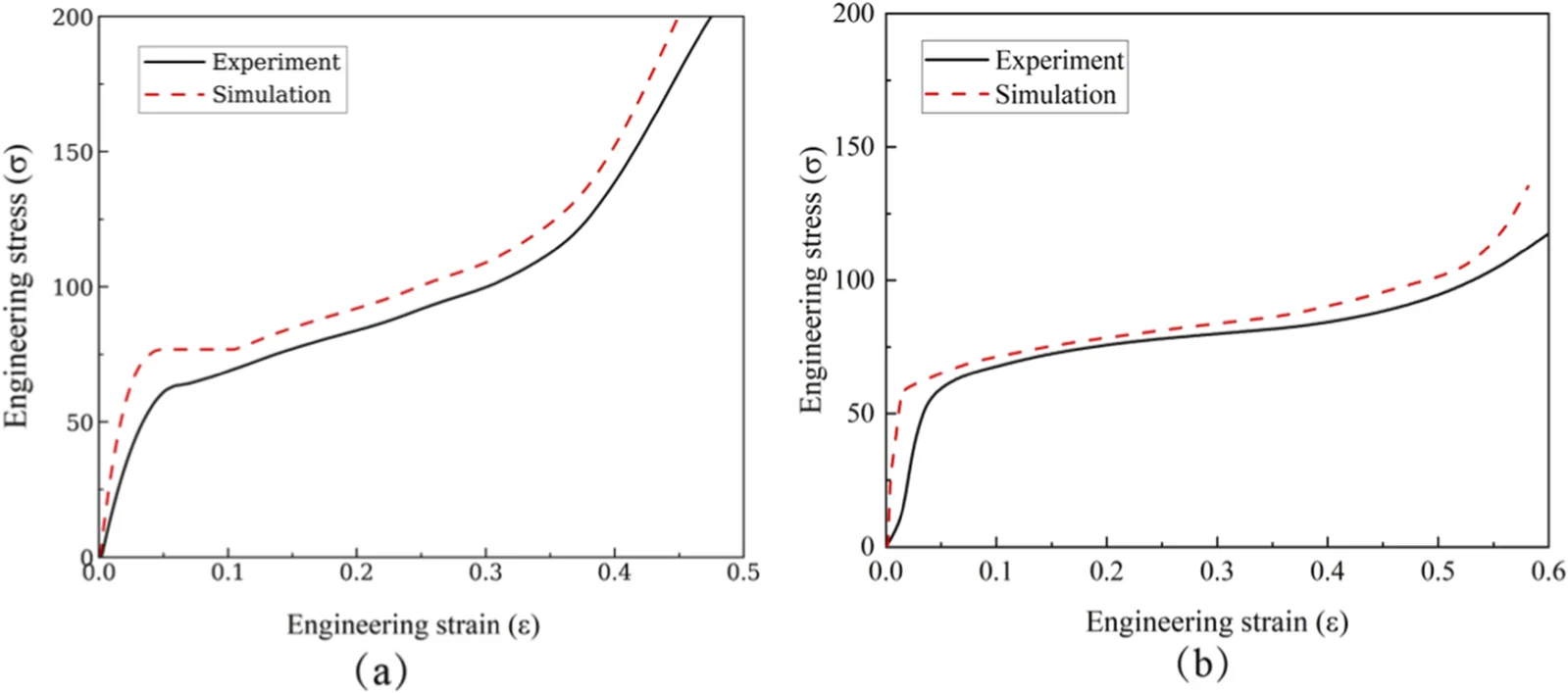
Comparison between experimental and FE-predicted stress–strain curves of the 70% uniform diamond structure under **(a)** 0° and **(b)** 45° loading.

It should be noted that direct experimental validation was not performed for the axial- and transverse-gradient structures. Therefore, the mechanical responses of the gradient configurations reported in this study should be regarded as numerical predictions based on the experimentally validated FE framework. Further experimental verification of representative gradient structures is required in future work.

Although the numerical and experimental stress–strain curves exhibit good agreement in the overall response, some discrepancies remain, particularly in the onset of the stress increase and the local stress level during compression. The numerical response occurs slightly earlier than the experimental response. This difference can be attributed to several factors. First, the FE model is based on an idealized geometry, whereas the additively manufactured specimens inevitably contain geometric imperfections, local wall-thickness variations, and surface roughness. These manufacturing-induced deviations can reduce the effective stiffness and delay the load-bearing response of the experimental specimens. Second, the Ti-6Al-4V constitutive parameters used in the simulations were obtained from literature data and may not fully reproduce the mechanical properties of the printed material. Third, the numerical contact formulation represents an idealized interaction between the specimen and compression platens, whereas small misalignment, local frictional variations, and surface irregularities may occur during the experiments. Despite these differences, the FE model successfully reproduces the overall elastic response, plateau behavior, and densification trend of the experiments, indicating that the model is suitable for the comparative analyses performed in this study. Therefore, the present FE framework is primarily intended for comparative evaluation of the effects of loading orientation and porosity distribution rather than exact reproduction of every local feature of the experimental curves.

### 3.2 Analysis of the energy absorption behavior

On uniform Diamond structures with load-bearing angles of 0°, 15°, 30°, 45° and 70% porosity, quasi-static compression tests were performed. In parallel, on transverse gradient and axial gradient Diamond structures with the same load-bearing angles quasi-static compression simulations were carried out. Each homogeneous structure was tested in triplicate. Given the minimal errors among the three sets of experiments, the average values were used to plot the curves. When the load-bearing angle is 45°, the uniform Diamond structure’s stress plateau stage becomes noticeably shorter and the densification phenomenon occurs earlier (Figure 7a). At a loading angle of 30°, the uniform Diamond structure exhibits enhanced energy-absorption behavior, characterized by a prolonged stress plateau and an increased plateau stress, resulting in improved energy-absorption performance. The simulation results of the axial gradient and transverse gradient structures were transformed into stress-strain curves. After applying the axial gradient, the stress of the structure increases rapidly during the compression process and does not exhibit a clear stress plateau region (Figure 7b). In addition, as the strain increases, minor fluctuations appear in the stress–strain curve, which indicates that the structure suffers from localized damage and failure during compression. The transverse gradient Diamond structure exhibits an obvious secondary energy absorption plateau feature at a load-bearing angle of 30° (Figure 7c), which is consistent with the observation of the homogeneous structure, which suggests that the transverse gradient did not have a significant effect on the internal stresses of the structure.

**FIGURE 7:**
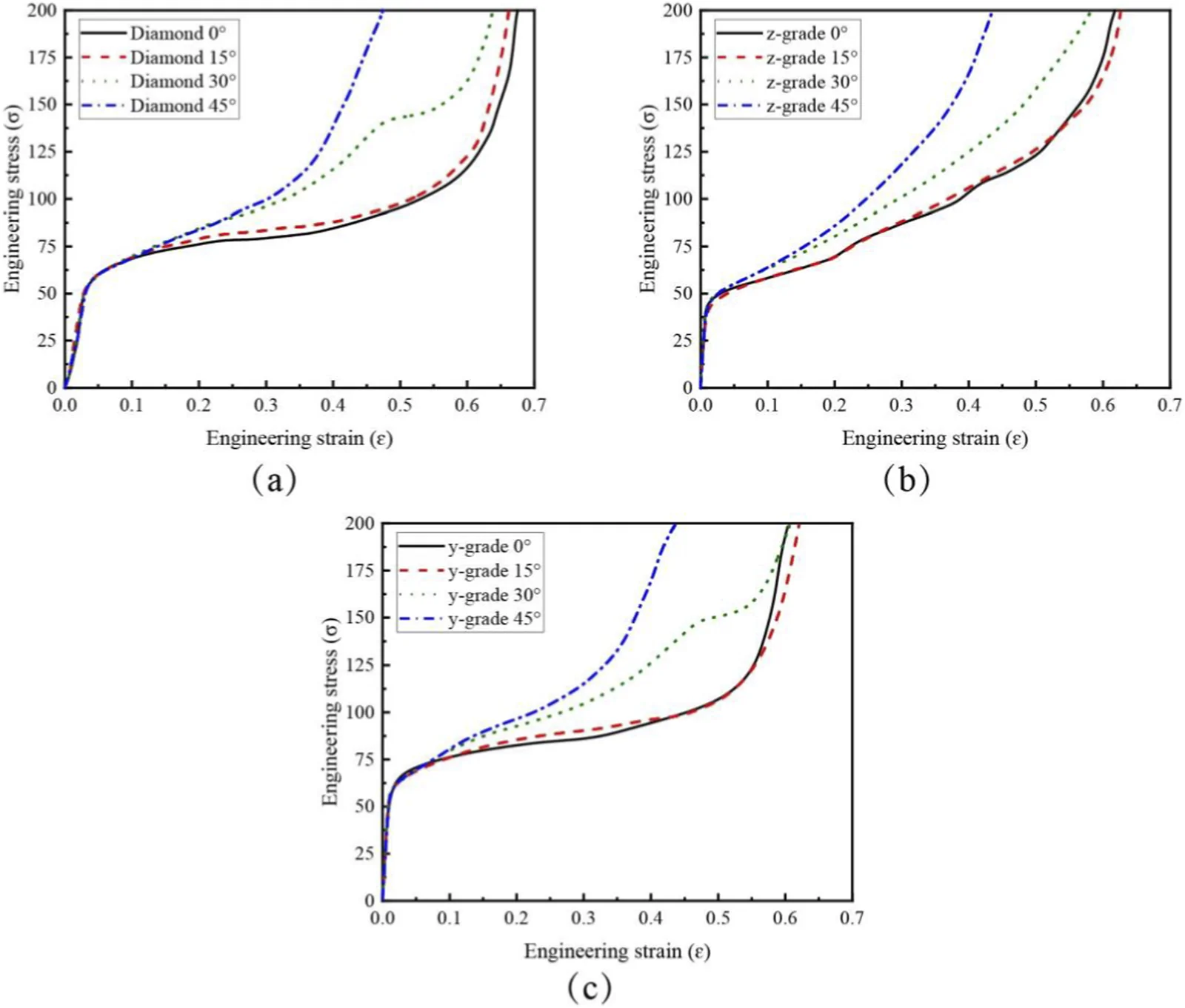
Stress-strain curves for **(a)** uniform diamond, **(b)** axial gradient diamond and **(c)** transverse gradient diamond structures. The experimental curves of the uniform diamond structures represent the mean stress–strain responses obtained from three independent tests (n = 3).

Figure 3 demonstrates the energy absorption efficiency curve of the Diamond structure. As shown in Figure 3a, the energy-absorption efficiency of the homogeneous Diamond structure shows a gradual increase during compression and reaches a maximum value at the densification point. When the structure enters the densification stage, the energy absorption efficiency decreases rapidly, this suggests that the structure ceases to absorb additional energy instead transmits it to the other side through the already densified structure. It is noteworthy that at the load-bearing angle of 45° the maximum energy absorption efficiency of the homogeneous Diamond structure decreases significantly, which appears to be associated with the shortened stress plateau stage. As shown in Figure 3b, the energy absorption efficiency curves of the axial gradient Diamond structure indicate that its maximum energy absorption efficiency is lower than 35% at different load-bearing angles, while the maximum energy absorption efficiency of the uniform structure is about 40%. This indicates that the axial gradient Diamond structure’s energy conversion efficiency is reduced as compared to the uniform structure. As shown in Figure 3c, the maximum energy absorption efficiencies of the transverse Diamond structures are all approximately 40%, which is consistent with the performance of the uniform structure. This finding suggests that under quasi-static compression conditions, the structures are relatively insensitive to the transverse gradient, and their internal stresses and energy-absorption efficiencies are almost indistinguishable from those of the uniform structures.

Figure 4 demonstrates the energy absorption behavior of uniform, axial gradient, and transverse gradient Diamond structures under various load-bearing angles. The load-bearing angle shows a significant impact on SEA of the Diamond structure. When the load-bearing angles were 0° and 15°, the SEA of the homogeneous Diamond structure varied with a small magnitude of about 6 kJ/kg however, when the load-bearing angle was increased to 30°, the SEA of the homogeneous structure was significantly increased by 33% to about 8 kJ/kg in this case, the dense strain of the homogeneous structure did not change significantly, but the plateau stress was significantly increased to about 105 MPa. As shown in Figure 7a, a secondary energy-absorbing plateau appeared in the structure at this point, which led to the increase in SEA. When the load-bearing angle was further increased to 45°, the dense strain of the homogeneous structure occurred earlier at about 0.37, while the plateau stress did not change significantly, which led to a decrease in SEA to 4 kJ/kg.

After applying the gradient design, the difference in the response of the structure at each loading angle is small, indicating that the gradient design mainly affects the energy absorption performance of the structure under frontal loading, while the sensitivity to the loading angle is relatively low. Compared with the uniform structure, the SEA of the axial gradient diamond structure is slightly improved at all loading angles. This is due to the fact that the dense strain of the axial gradient structure is not significantly changed, but the plateau stress is significantly increased, which leads to the improvement of the SEA. Since the porosity of the uniform diamond structure is 70%, while the gradient interval of the porosity in the transverse direction ranges from 60% to 80%, and the plateau stress of the transverse gradient diamond structure is basically the same as that of the uniform structure, it can be concluded that the plateau stress of the transverse gradient structure is equal to the plateau stress of the uniform diamond structure corresponding to the central porosity in this gradient interval. On the contrary, the plateau stress of the transverse-gradient diamond structure does not change significantly, but the dense strain occurs in advance, so the SEA of the structure shows a certain decreasing trend.

### 3.3 Analysis of the deformation behavior

Under compressive loading, the deformation behavior of TPMS structures is mainly manifested in two modes: tensile-dominated and bending-dominated, both of which strongly influence their mechanical response and energy absorption behavior (Qiu et al., 2024). The tensile-dominated TPMS structure shows a high initial load-bearing capacity in the early stage of compression, however, with the increasing degree of deformation, its load-bearing capacity gradually decreases, and finally enters the densification stage. In contrast, the flexure-dominated TPMS structure has a longer stress plateau region and exhibits better energy absorption, but its overall strength and stiffness are relatively low (Deshpande et al., 2001). Related studies have shown that tensile-dominated structures are preferable for applications requiring high initial load-bearing capacity, while bending-dominated structures are better suited for sustained and stable energy dissipation (Ashby, 2006). These properties provide an important theoretical basis for the optimal design of TPMS structures in different application scenarios.

In order to investigate the deformation pattern of the structure in compression, the stress clouds of the structure under 15%, 30%, 45% and 60% strain moments in compression are extracted in this paper. The legend scale of the simulation results is set uniformly in order to show the internal stresses of the structure more clearly at various load-bearing angles.

The uniform Diamond structure exhibits variable deformation behaviors and stress distributions under various load-bearing angles, manifesting distinct patterns of transverse displacement, extrusion, and strain hardening. These phenomena significantly influence the structure’s energy absorbing performance. Figure 8 shows the stress cloud diagram of the uniform Diamond structure. When the load-bearing angle was 0°, the uniform Diamond structure did not show obvious transverse displacement deformation; when the load-bearing angle was 15°, the structure showed only slight transverse deformation, and the stress distribution in its deformation process was relatively uniform, with no formation of obvious transverse bands or local extrusion deformation. However, when the load-bearing angle was 30°, the structure not only showed obvious transverse displacement in the compression process, but also showed the phenomenon of layer-by-layer extrusion, accompanied by extrusion deformation on one side and tensile deformation on the other side. As compression proceeds, the structure with a load-bearing angle of 30° collapses layer by layer until the extrusion deformation of the last layer is completed, and the internal stress of the entire structure begins to increase, leading to the formation of a secondary plateau. Following the secondary plateau action period, the entire structure gradually enters into a dense state. This process helps to increase the structure’s average plateau stress value, thus allowing the energy-absorbing capabilities of the structure to fully develop. On the contrary, the structure shows limited deformation in the vertical direction (Z-axis direction) at the load-bearing angle of 45°, and the structure enters the dense state almost immediately. This indicates a severe strain hardening phenomenon in the structure. The pronounced secondary plateau observed at a loading angle of 30° can be attributed to the interaction between the loading direction and the orientation-dependent load-transfer pathways of the Diamond architecture. At 30°, the applied load is redistributed among multiple inclined load-bearing regions, resulting in a more distinct sequential activation of different structural parts during compression. Following the initial localized collapse, additional regions progressively participate in load bearing, which temporarily stabilizes the macroscopic stress response and gives rise to the secondary plateau.

**FIGURE 8:**
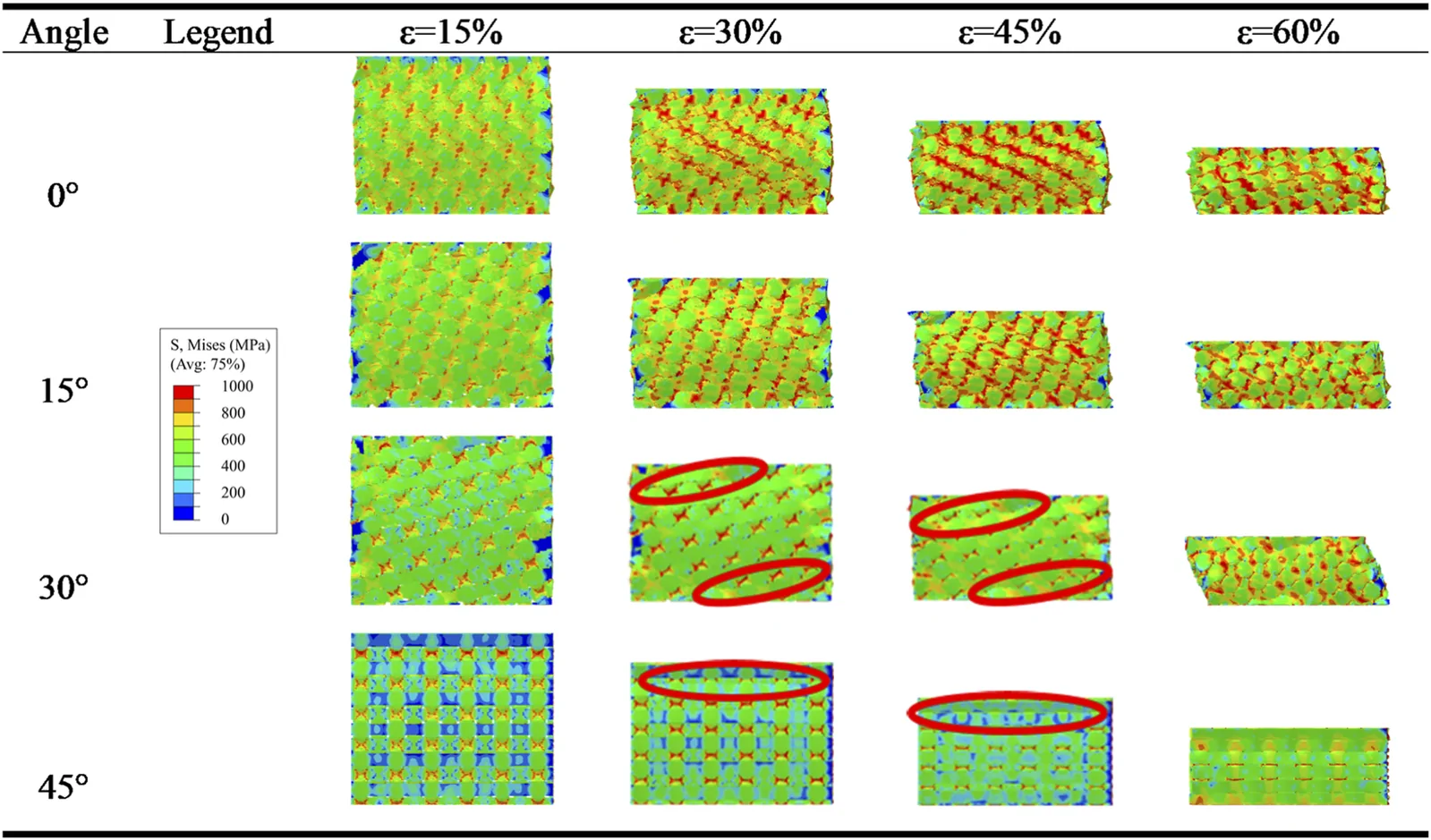
FE simulation results of the uniform diamond structure.

In comparison, the 15° configuration remains closer to the 0° loading condition and exhibits a more continuous collapse process without a pronounced transition between different load-bearing stages. At 45°, stronger deformation localization and earlier densification shorten the effective plateau region. Therefore, the secondary plateau observed at 30° is interpreted as a consequence of staged load redistribution and progressive structural engagement within the Diamond architecture rather than as a general feature of all oblique loading conditions.

It should be noted that this interpretation is based on the observed stress redistribution and deformation patterns. A quantitative statistical analysis of individual load-bearing member orientations and their contributions was not performed in the present study.

The axial gradient diamond structure demonstrates considerable stress inhomogeneity during compression. Stress concentrates on the side with greater porosity due to its diminished load-bearing capacity, resulting in localized deformation and progressive structural collapse. Figure 9 illustrates the stress cloud of the axial gradient Diamond structure. During the compression process of the axial gradient Diamond structure, the stress distribution inside the structure shows obvious inhomogeneity, and the stress tends to concentrate preferentially on the side with larger porosity. This phenomenon is rooted in the fact that the region with larger porosity is relatively weak and its node connectivity is poor, resulting in a lower load-bearing capacity. As a result, this side is more likely to yield under external forces. As compression proceeds, localized deformation first develops in the high-porosity region and subsequently progresses layer by layer toward the low-porosity region, eventually affecting the uppermost side with smaller porosity.

**FIGURE 9:**
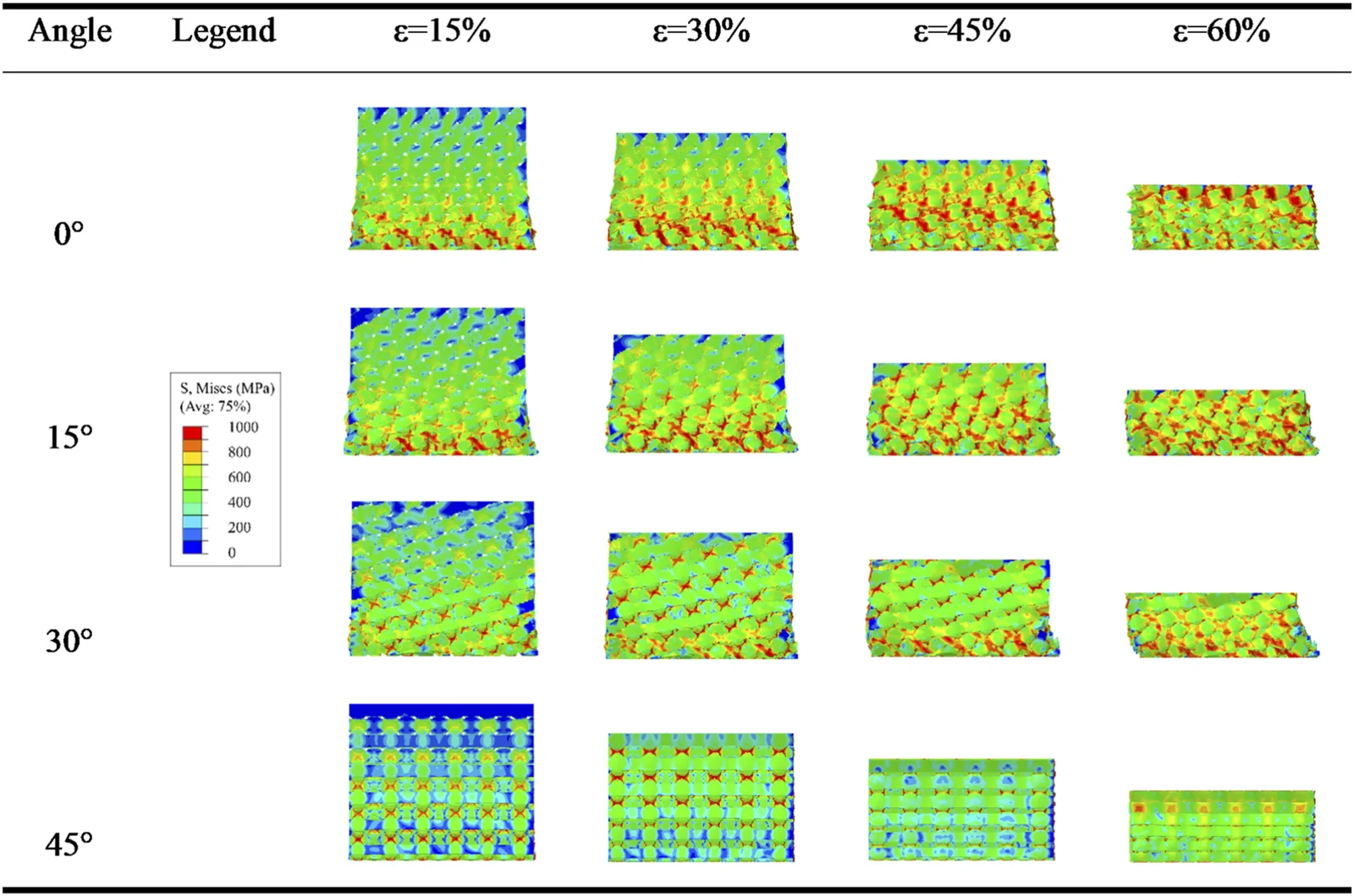
FE simulation results of the axial gradient diamond structure.

The transverse gradient Diamond structure demonstrates unique mechanical behaviors during quasi-static compression, as indicated by distinct stress distribution and deformation patterns that are influenced by its gradient design. Figure 10 illustrates the stress cloud of the transverse gradient Diamond structure. During quasi-static compression, the transverse gradient Diamond structure exhibits unique stress distribution characteristics. Specifically, the internal stress on the less porous side of the structure is always higher than that on the more porous side. This phenomenon is mainly attributed to the fact that the side with lower porosity has a higher load-bearing capacity and thus absorbs more compressive loads, resulting in the first concentration of stresses on that side. However, the side with higher porosity also shares some of the load when loaded, which changes the overall load carrying capacity of the structure due to the presence of a linear gradient.

**FIGURE 10:**
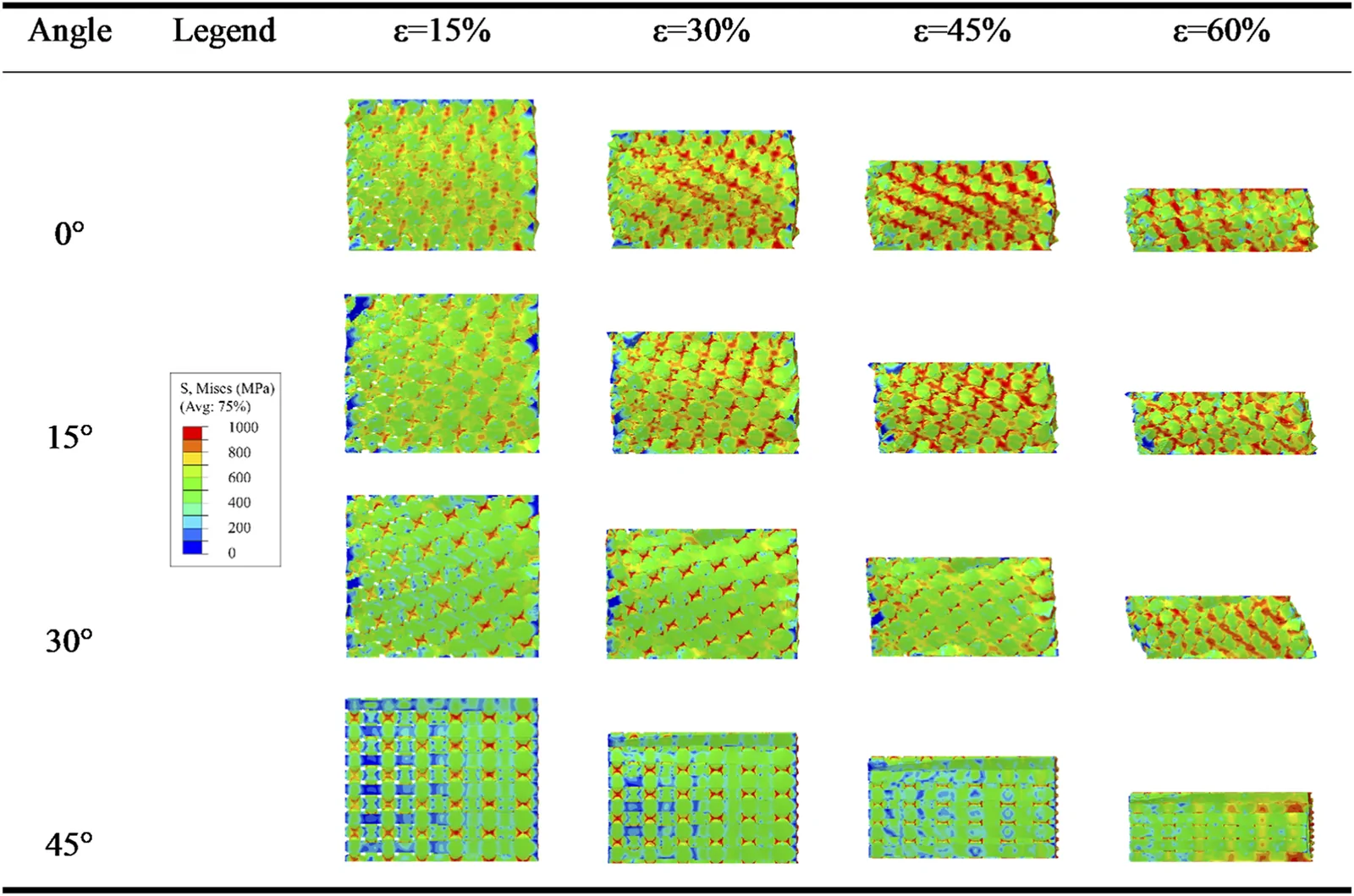
FE simulation results of the transverse gradient diamond structure.

The transverse gradient Diamond structure undergoes progressive extrusion deformation and structural collapse developing from the lower-porosity region, which then propagates to the higher porosity side, leading to overall extrusion failure and dense state entry. This early densification is primarily driven by the lower porosity side. During this process, the structure exhibits lateral displacement at different load bearing angles, but the differences are not significant compared to uniform and axial gradient Diamond structures, indicating that the deformation behavior is relatively insensitive to gradient changes.

## 4 Conclusion

In this paper, uniform Diamond structures and Diamond structures with porosities varying along the axial and transverse directions, respectively, under different load-bearing angles are generated. Through finite element quasi-static compression simulations, the changes in energy absorption properties of Diamond structures under different loading-bearing angles with porosities linearly graded along the axial and transverse directions, respectively, were analyzed, and the damage and deformation modes during the compression process were also examined. The main conclusions are as follows.

1. Axial gradient Diamond structures exhibit enhanced load-bearing capacity and energy absorption performance but with slightly reduced energy absorption efficiency.
2. Transverse gradient Diamond structures enter the dense state earlier, leading to decreased SEA.
3. The axial gradient Diamond structure increases the SEA by increasing the plateau stress.
4. The plateau stresses of transverse gradient Diamond structures are comparable to those of uniform structures with intermediate porosity. The lateral displacements of different structures show no significant differences at various load-bearing angles.

From a biomedical perspective, the present study provides mechanical design insights into the effects of loading orientation and porosity-gradient direction on the compressive and energy-absorption behavior of Ti-6Al-4V Diamond structures. However, the suitability of these structures for orthopedic or bone-tissue-engineering applications cannot be evaluated solely on the basis of compressive and energy-absorption performance. Other important factors, including pore interconnectivity, permeability, effective stiffness, fatigue resistance, biocompatibility, osseointegration, and vascularization, were not investigated in the present study. Therefore, the current results should be interpreted as a mechanical-design reference rather than direct evidence of biomedical suitability. Further experimental, biological, and long-term mechanical studies are required before application-specific conclusions can be drawn.

## Data Availability

The original contributions presented in the study are included in the article/supplementary material, further inquiries can be directed to the corresponding author.
